# Color Doppler carotid resistance, pulsatility, and aortic oscillometry indices in giant cell arteritis: insights from the VASCARD cohort

**DOI:** 10.1186/s13075-026-03779-w

**Published:** 2026-03-28

**Authors:** Konstantinos Triantafyllias, Anna-Lena Zang, Andreas V Goules, Elena K Joerns, Muthuraman Muthuraman, Andreas Schwarting

**Affiliations:** 1 Department of Internal Medicine I, Division of Rheumatology and Clinical Immunology, Johannes Gutenberg University Medical Center, Mainz, Germany; 2 Department of Pathophysiology, School of Medicine, National and Kapodistrian University of Athens, Athens, Greece; 3 Division of Rheumatology, Department of Internal Medicine, Mayo Clinic, Rochester, MN USA; 4 Department of Neurology, University Hospital Würzburg, Würzburg, Germany; 5 Department of Rheumatology, Acute Rheumatology Center, Bad Kreuznach, Germany

**Keywords:** Giant cell arteritis, Aortic stiffness, Pulse wave velocity, Atherosclerosis, Carotid sonography, Pulsatility index, Resistance index

## Abstract

**Background:**

To assess a combination of novel color Doppler ultrasound (CDUS), greyscale ultrasound (GSUS), and oscillometric indices of macroangiopathy and (CV) risk in patients with giant cell arteritis (GCA). Additionally, to explore the relationships between these imaging markers and both patient-specific and disease-related characteristics, as well as traditional CV risk factors.

**Methods:**

CDUS was performed to evaluate arterial compliance markers, specifically the resistance (RI) and pulsatility (PI) indices, in both the common (CCA) and internal carotid artery (ICA) of GCA patients and healthy controls. GSUS examinations were conducted to measure carotid intima-media-thickness (cIMT), identify plaques, and quantify cumulative carotid calcification surface. Oscillometry was utilized to determine aortic stiffness via carotid-femoral pulse-wave velocity (cfPWV).

**Results:**

Sixty-six GCA patients and 93 healthy subjects were included. Patients showed significantly higher cfPWV (p_adj_ <0.001), cIMT (p_adj_ =0.037), CCA-PI (p_adj_ =0.009), CCA-RI (p_adj_=0.019), and plaque-area (*p* < 0.001) compared with controls. cfPWV correlated with traditional CV risk factors, like age (rho = 0.330, *p* < 0.009), mean-arterial-pressure (rho = 0.257, *p* = 0.044), and cholesterol (rho = 0.318, *p* = 0.017). CCA-PI and -RI were lower in patients receiving immunosuppressive therapy [1.5 (1.33–2.04) vs. 1.95 (1.72–2.29); 0.74 (0.68–0.82) vs. 0.80 (0.76–0.85), both; *p* < 0.05], and CCA-RI was predicted by erythrocyte-sedimentation-rate (rho = 0.343, *p* = 0.044). cIMT correlated with age (*r* = 0.619, *p* < 0.001) and plaque area (rho = 0.305, *p* = 0.047).

**Conclusion:**

GCA patients demonstrated increased carotid pulsatility, resistance, atherosclerosis, and aortic stiffness compared to controls. Moreover, key predictors of impaired CV and cerebrovascular surrogates were identified. The combined assessment of CDUS and GSUS could provide a more thorough evaluation of the arterial tree, thereby enhancing the overall assessment of macroangiopathy.

**Trial registration:**

DRKS00031470.

**Supplementary Information:**

The online version contains supplementary material available at 10.1186/s13075-026-03779-w.

## Introduction

Vasculitis refers to a group of disorders characterized by inflammation of the blood vessels, leading to a wide range of clinical manifestations, depending on the affected vascular territory and organ involvement [1]. The most common primary systemic vasculitis is giant cell arteritis (GCA), a granulomatous inflammation typically affecting large vessels like the aorta and its main branches, with a particular predilection for the carotid, vertebral, and temporal arteries [1, 2]. GCA occurs in persons over the age of 50 and is more frequent in females than males [1, 2]. As a result of vessel inflammation, GCA presents with a wide spectrum of symptoms, ranging from mild manifestations such as polymyalgia rheumatica to more severe complications, with visual impairment being the most common and serious outcome [2].

Common comorbidities in patients with GCA include cardiovascular (CV) conditions such as ischemic heart disease, venous thromboembolism, and cerebrovascular (CVB) events, along with diabetes mellitus and severe infections [2, 3]. Regarding CV disease, it is known that chronic systemic inflammation can impair endothelial function and arterial elasticity, leading to accelerated atherosclerosis and increased CV risk [4, 5]. This is primarily caused by inflammation-induced damage to the endothelium, leading to the infiltration of inflammatory cells (such as macrophages, dendritic cells, and T-lymphocytes) into the arterial wall [6]. This process also involves the deposition of lipids, particularly LDL, the formation of foam cells, and ultimately atherosclerotic plaque development [6, 7].

Importantly, the leading causes of mortality in GCA are CV events [8, 9]. This has been described in various meta-analyses, like that of Lee et al., who assessed standardized mortality ratios (SMRs) in 1972 GCA patients (SMR 1.312, 95%CI 1.136–1.516, *p* < 0.001) [8]. Moreover, Uddhammar et al. found increased CV mortality ratios in both female and male patients with GCA [SMR = 149 (95%CI 118–189) and 158 (95%CI 112–224)] when examining a cohort of 136 GCA and 35 PMR patients for approximately 20 years [9].

Although CV risk, CV morbidity, and mortality rates are increased in GCA patients, there is limited data available on how to classify and assess these risks. CV screening methods which have been validated in the general population, such as the Systematic Coronary Risk Evaluation (SCORE) proposed by the European Society of Cardiology (ESC), potentially underestimate risk of CV disease in patients with autoimmune rheumatic diseases, since they do not account for the effects of chronic inflammation and disease-associated aspects (e.g. glucocorticoids, reduced physical fitness, etc.) [10]. Therefore, there is a necessity for additional CV markers that would assist CV screening and improve angiopathy assessment in GCA.

Several non-invasive surrogate CV diagnostic methods have demonstrated strong diagnostic value both in the general population and across various rheumatic diseases [11–16]. For instance, carotid-femoral pulse wave velocity (cfPWV), considered the gold standard for assessing aortic stiffness, has demonstrated high predictive performance for CV events in the general population, with values > 10 m/s indicating an increased CV risk and presence of end-organ disease [12, 13]. In addition, carotid ultrasound, especially cIMT, has proven to be a reliable tool for the assessment of carotid atherosclerosis [14–16]. In the last years, our research group and others have examined the diagnostic role of several CV risk surrogates in patients with different rheumatic diseases like RA [17], psoriatic arthritis [18], systemic lupus erythematosus [19], mixed connective tissue disease [20], myositis [21] and antisynthetase syndrome [10], as well as fibromyalgia [22].

However, data regarding CV surrogates are limited in patients with GCA and/or included small-sized cohorts [23]. Therefore, this pilot study aimed to examine for the first time a wide panel of CV surrogates, including the gold standard oscillometric marker of aortic stiffness, color Doppler indices of vascular compliance and vascular resistance, and well-known carotid sonography markers of atherosclerosis in comparison to healthy controls. Additionally, we sought to evaluate associations of cfPWV/carotid ultrasound indices with disease characteristics and patient-associated parameters, as well as traditional CV risk factors in one of the largest GCA cohorts examined.

## Methods

### Study population

Recruitment of patients took place in the context of the ‘‘VASculitis associated CARDiovascular risk (VASCARD)’’ cohort study, a part of the German CARD cohorts, comprising 66 consecutive GCA inpatients being treated in our Rheumatology Center and fulfilling the ACR/EULAR criteria [24].

A control group of 93 individuals, comprised of hospital staff and healthy volunteers from their acquaintances without underlying inflammatory diseases, was recruited through an open call for voluntary study participation. All patients and controls underwent cfPWV assessments. The examination was standardized in accordance with the recommendations of the expert consensus document on arterial stiffness measurement [12]. Participants rested in the supine position for at least ten minutes before the measurement began. All measurements were performed around midday to minimize circadian variation. The first antihypertensive medication of the day was taken according to the medication schedule in the morning. During the measurements, participants were instructed not to speak.

Additionally, subgroups were examined using B-mode and Doppler ultrasound of the common carotid (CCA) and internal carotid arteries (ICA). Exclusion criteria were age < 50 years, body mass index (BMI) > 45 kg/m^2^, malignancy, pregnancy, and end-stage renal failure. Patients provided informed consent, and the study protocol was reviewed and approved by the ethics committee of the Rhineland-Palatinate medical board, Germany, in accordance with the principles of the Helsinki Declaration (approval number: 13762_2).

### Data collection

For both groups, we collected epidemiological and anthropometric data, like age, sex, BMI, and disease duration. Laboratory parameters such as inflammatory markers (erythrocyte sedimentation rate [ESR], C-reactive protein [CRP]), lipids, renal parameters, and various traditional CV risk factors like arterial hypertension, use of antihypertensive drugs, statins, diabetes mellitus, and nicotine use were also documented. To address the limitations of inflammatory markers alone, disease activity was defined based on elevated ESR and/or CRP, supplemented by evidence of vascular inflammation on imaging modalities (MRI, CT, or vascular ultrasound) when available. In the patient group, we collected clinically relevant characteristics such as CV events (e.g., heart attack, stroke, atrial fibrillation, TVT, pulmonary embolism), headaches, or eye involvement prior to analysis. In addition, we recorded the use of disease-modifying antirheumatic drugs (DMARDs), especially the class, dosage, and duration, as well as the use of glucocorticoids.

### cfPWV and sonography

Carotid-femoral PWV (cfPWV) was assessed non-invasively using a Vicorder device from SMT Medical (Wuerzburg, Germany), as described previously [12, 25, 26]. The cfPWV examination protocol was carried out according to the manufacturer’s instructions and the expert consensus on arterial stiffness [12]. The device calculated the velocity of pulse waves passing between two points through the wall of the carotid artery and the femoral artery by dividing (0.8 x) the traveled pulse wave distance by the pulse transit-time [12]. We documented the average of 3 measurements, and cfPWV values > 10 m/s were regarded as a sign of increased CV risk [12].

Carotid ultrasonography was performed by an experienced examiner (KT, certified trainer of the German Society for Ultrasound in Medicine; DEGUM) under blinded conditions. cIMT and plaque presence in the internal and external carotid arteries were analyzed using a MyLab 9-US device (Esaote^®^) with a 4–15 MHz linear transducer operating at a maximal frequency. Plaque presence (localized thickenings > 1.2 mm) was bilaterally examined in the CCA and the carotid bulbs, and the plaque area was documented in cm^2^ for further analysis. B-mode cIMT was measured in the end-diastolic phase at three consecutive locations of the bilateral CCA, approximately 1 cm proximal to the carotid bulb. The maximum value of these measurements was used for statistical analysis. Subclinical carotid atherosclerosis (SCA) was defined as cIMT > 0.9 mm and/or presence of ≥ 1 plaque(s) [27].

CDUS examinations of proximal ICA and mid-CCA were performed on both sides using transverse and longitudinal planes. Doppler analysis was used to assess peak systolic (PSV) and end-diastolic velocity (EDV). A pulsed wave flow spectrum was recorded after 5 s of several identical waveforms. Using the Pourcelot [RI = (PSV – EDV)/PSV] and Gosling’s formulas [(PSV – EDV)/mean flow velocity (MFV)], the device automatically calculated resistance (RI) and pulsatility (PI) indices. The maximum vessel indices were also recorded for statistical purposes.

### Statistical analysis

The normality of variable distribution was assessed using the Shapiro-Wilk test. Descriptive statistics included mean ± standard deviation (SD) for normally distributed and median with 25th/75th percentiles for non-normally distributed variables. For categorical variables, relative (%) and absolute (n) frequencies were determined. The correlations between the surrogate parameters and continuous variables were determined using Spearman’s (non-normal distribution) and Pearson’s (normal distribution) correlation coefficients. The comparison between the patient group and the control group was carried out for continuous variables using the t-test or the Mann-Whitney U test, according to the distribution. The categorical variables were compared using the chi^2^ test. The significance level was set at a *p*-value of < 0.05.

Binary logistic regression was performed with significant differences between patients and controls to adjust for confounding. We adjusted for five covariates that we chose a priori and have been shown to associate with CV risk, including age, BMI, diabetes, nicotine use, and arterial hypertension. Moreover, we included sex as a sixth covariate according to common practice regarding adjustment analyses and mean arterial pressure (MAP) for the case of cfPWV, given that MAP is a known confounder of cfPWV. The sample size was calculated to ensure adequate power for logistic regression. Unadjusted and adjusted analyses were performed using IBM SPSS^®^ 27.0 software.

To address non-randomization and control for confounding factors (age, sex, BMI, nicotine use, diabetes, and arterial hypertension), we performed Bayesian propensity score matching (PSM) between groups. Propensity scores were estimated as previously described [28]. We conducted PSM using the Bayesian spatial PSM toolbox in RStudio (v1.1.456) (https://sejdemyr.github.io/r-tutorials/statistics/tutorial8.html). In the first step, propensity scores were derived via Bayesian logistic regression. As Bayesian networks are generative models, the joint probability distribution was defined using Bayes’ theorem, where the posterior is the product of the likelihood and prior. Since exact posterior distributions are often intractable, we approximated them using the No-U-Turn Sampler (NUTS) within the Hamiltonian Monte Carlo framework. In the second step, we matched subjects based on propensity score distances using caliper matching [29]. After matching, we performed log-rank testing.

## Results

cfPWV was assessed in 62 GCA patients and 88 healthy controls. Additionally, Doppler and B-mode sonography of CCA and ICA were performed in subgroups consisting of 47 patients and 41 controls, respectively. The descriptive characteristics and the differences between the controls and GCA patients are presented in Tables 1 and 2.

**Table 1:** Descriptive characteristics by group (cfPWV)

	Controls (*n = 88)*	Patients (*n = 62)*	Significance
Age (years)	57.5 (54.25-62,00)	70.5 (61.75-78)	< 0.001***
Sex *(female)*	74 (84.1%)	49 (79.0%)	0.427
Nicotine use *(smokers)*	12 (16.2%)	9 (17.3%)	0.258
Hypertension **(***yes)*	25 (33.8%)	50 (80.6%)	< 0.001***
Antihypertensive drugs (*yes)*	25 (33.8%)	43 (69.4%)	< 0.001***
Diabetes *(yes)*	1 (1.4%)	9 (14.5%)	0.004**
BMI *(kg/m2)*	25.95 (22.5–28.6)	25 (22.8–28.8)	0.376
MAP ¶ *(mmHg)*	95.72 ± (10.43)	101.56 ± (12.45)	0.002**
Heart rate¶ *(/min)*	66.6 ± (10.21)	74.62 ± (9.86)	< 0.001***
cfPWV *(m/s)*	7.57 (6.89–8.16)	9.91 (8.62–10.80)	< 0.001*** < 0.001***¥ <0.002**¥¥
CRP *(yes)*	4 (12.9%)	26 (47.3%)	0.001**
Cholesterol ¶ *(mg/dl)*	218.44 ± (42.184)	219 (184.5-241.75)	0.823
HDL¶ *(mg/dl)*	66.9 ± (19.98)	66.79 ± (22.77)	0.931
LDL ¶ *(mg/dl)*	135.90 ± (36.78)	133.5 ± (69.56)	0.318
Kreatinin¶ (*mg/dl)*	0.79 ± (0.133)	0.89 ± (0.2261)	0.071
DMARDS *(yes)*	-	31 (50%)	-
DMARDS duration *(months)*	-	8.0 ± 7.47	-
Azathioprine dose (*n = 2) (mg)*	-	Both; 50	-
Tocilizumab dose (*n = 16)(mg)*	-	All; 162	-
Methotrexate dose (*n = 11)(mg)*	-	12.7 ± 4.80	-
Baricitinib dose (*n = 1) (mg)*	-	2	-
Etanercept dose (*n = 1) (mg)*	-	50	-
Leflunomide dose (*n = 1) (mg)*	-	10	-
Glucocorticoids *(mg)*	-	7.25 (2.88–23.75)	-
Glucocorticoids *(yes)*	-	50 (80.6%)	-
Statins (yes)	3 (4.1%)	19 (33.9%)	< 0.001***
Vision impairment *(yes)*	-	23 (37.1%)	-
Headache (yes)	-	31 (51.7%)	-
Cardiovascular events *(yes)*		21 (33,9%)	
Disease duration *(months)*	-	10 (5–33)	-

All non-normal distributions: presentation as median (interquartile range)

Mann-Whitney-U test ( non-normal distribution; median, interquartile-range)

*BMI* body mass index, *MAP* mean arterial pressure, *cfPWV* carotid-femoral pulse wave velocity, *CRP* C-reactive protein, *HDL* high-density lipoprotein, *LDL* low-density lipoprotein, *DMARDs* disease-modifying anti-rheumatic drugs

t-test (normal distribution; mean ± SD)

¶normal distribution: presentation as mean ± standard deviation

** *p* < 0.01

*** *p* < 0.001

¥ = *p*-value adjusted for age, BMI, nicotine use, sex, diabetes, hypertension

¥¥ = *p*-value adjusted age, BMI, nicotine use, sex, diabetes, hypertension, and MAP

**Table 2:** Descriptive characteristics by group (Carotid-Sonography)

	Controls (*n = 41)*	Patients (*n = 47)*	Significance
Age (years)	57 (54–62)	71.0 (61–78)	< 0.001***
Sex *(female)*	34 (82.9%)	39 (83%)	0.995
Nicotine use *(smokers)*	7 (20%)	8 (20.0%)	0.788
Hypertension *(yes)*	14 (40%)	36 (76.6%)	< 0.001***
Antihypertensive drugs *(yes)*	14 (40%)	31 (66%)	0.019**
Diabetes *(yes)*	1 (2.9%)	8 (17.0%)	0.047*
BMI *(kg/m2)*	26.5 (23.40-29.16)	25.0(21.83–27.11)	0.020*
MAP *(mmHg)*	100 (93–103)	100 (92.5-106.7)	0.213
Heart rate¶ *(/min)*	68.20 ± (9.31)	74.62 ± (9.86)	0.024*
cfPWV(*m/s)*	7.91 (7.06–8.86)	9.84 (8.32–10.56)	< 0.001***
CRP *(yes)*	4 (28.6%)	20 (48.8%)	0.188
cIMT	0.83 (0.76–0.93)	0.99 (0.90–1.10)	< 0.001*** 0.037* ¥
CCA-PI	1.57 (1.34–1.73)	1.92 (1.58–2.22)	< 0.001*** 0.009** ¥
CCA-RI	0.73 (0.69–0.76)	0.79 (0.75–0.83)	< 0.001*** 0.019* ¥
ICA-PI	1.18 (0.94–1.42)	1.30 (1.10–1.61)	0.019* 0.132 ¥
ICA-RI ¶	0.66 (0.57–0.73)	0.70 ± (0.68)	0.010* 0.090 ¥
Plaque area *(cm^2)*	0.045 (0–0.1)	0.16 (0.07–0.37)	< 0.001***
Plaques ( yes)	18 (60%)	41 (89.1%)	0.003**
Cholesterol ¶ *(mg/dl)*	218.44 ± (42.18)	215.17 ± (50.16)	0.823
HDL¶ *(mg/dl)*	64.50 (53.25-77.00)	66.79 ± (22.77)	0.931
LDL ¶ *(mg/dl)*	135.90 ± (36.78)	118.48 ± (39.12)	0.318
Creatinine ¶ *(mg/dl)*	0.78± (0.12)	0.89 ± (0,28)	0.144
DMARDS *(yes)*	-	20 (42.5%)	-
DMARDS duration *(months)*	-	9.4 ± (7.64)	-
Azathioprine dose (*n = 2) (mg)*	-	Both; 50	-
Tocilizumab dose (*n = 12) (mg)*	-	All; 162	-
Methotrexate dose (*n = 4) (mg)*	-	11.3 ± (2.50)	-
Baricitinib dose (*n = 1) (mg)*	-	2	-
Leflunomide dose (*n = 1) (mg)*	-	10	-
Glucocorticoids (mg)	-	5 (1.36–16.88)	-
Glucocorticoids (yes)	-	35 (76.1%)	-
Statins (yes)	3 (8.6%)	14(34.1%)	0.008**
Vision impairment (yes)	-	13 (27.1%)	-
Headache (yes)	-	22 (48.9%)	-
Cardiovascular events *(yes)*		17 (36,2%)	
Disease duration *(months)*	-	11 (5–33)	-

non-normal distribution: presentation as median (interquartile range)

Mann-Whitney-U test ( non-normal distribution; median, interquartile-range)

*BMI* body-mass-index, *MAP* mean arterial pressure, *cfPWV* carotid-femoral pulse wave velocity, *CRP* C-reactive protein, *cIMT* carotid-intima-media-thickness, *CCA* common carotid artery, *ICA* internal carotid artery, *PI* pulsatility-index, *RI* resistance-index, *HDL* high-density lipoprotein, *LDL* low-density lipoprotein, *DMARDs* disease-modifying anti-rheumatic drugs

t-test (normal distribution; mean ± SD)

¶ = normal distribution: presentation as mean ± standard deviation

* *p* < 0.05

** *p* < 0.01

*** *p* < 0.001

¥ = *p*-value adjusted for age, BMI, gender, hypertension, nicotine, diabetes

### Association between group status (GCA vs. control): cfPWV and carotid-sonography

**Fig. 1:**
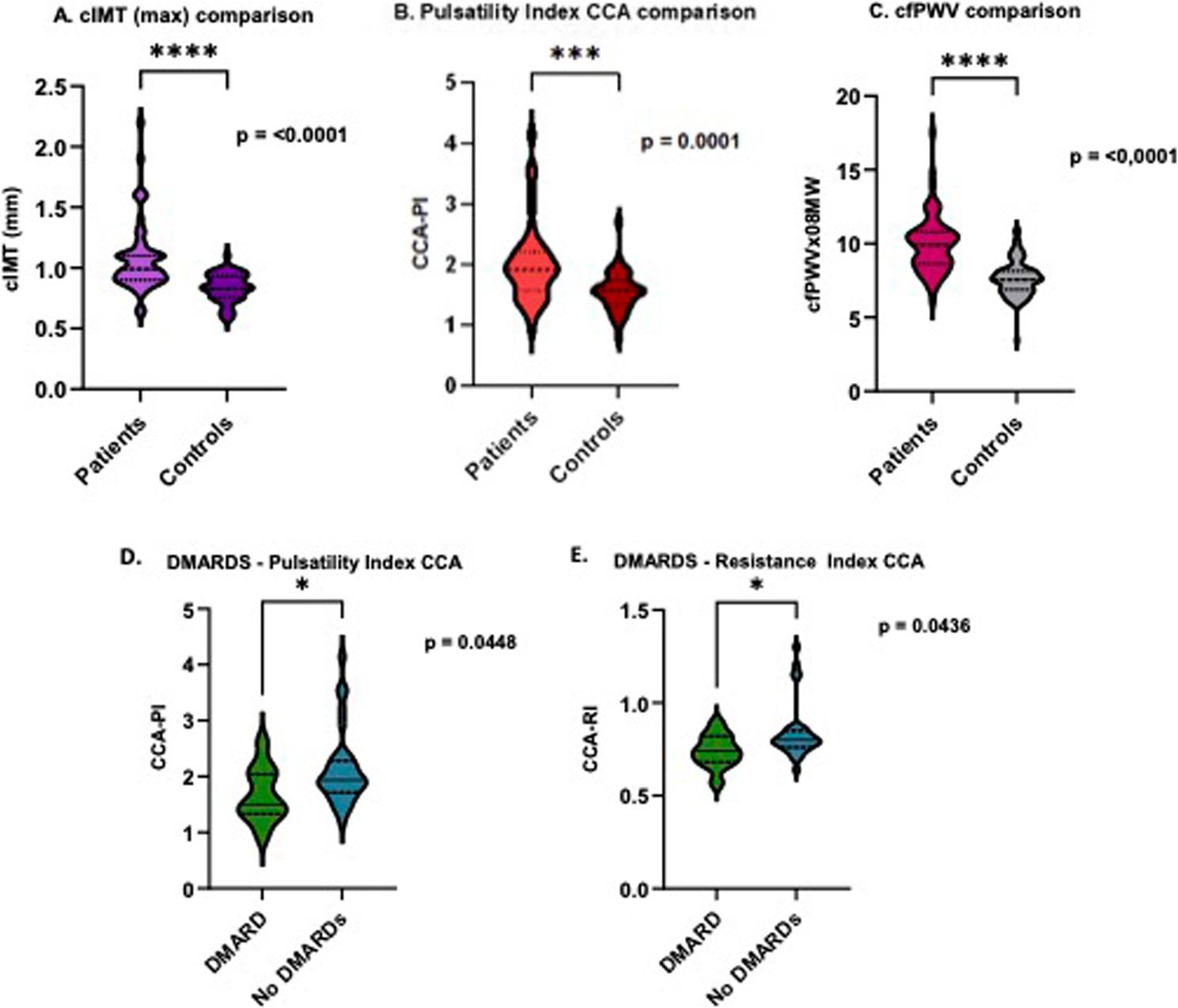
**A**, **B**, **C**: Comparison of surrogate CV markers (control vs. GCA patients); (**D**, **E**): Comparison of PI and RI of CCA (DMARD vs. no DMARDs). **A**. Comparison of carotid intima media thickness between patients and controls: *p* <0.001; **B**. Comparison of pulsatility index of the common carotid artery between patients and controls: *p* <0.001; **C**. Comparison of carotid-femoral pulse wave velocity between patients and controls: *p* <0.001; **D**. Comparison of pulsatility index of the common carotid artery between patients with DMARD vs. patients with no DMARDs: *p* = 0.0448. **E**. Comparison of resistance index of the common carotid artery between patients with DMARD vs. patients with no DMARDs: *p* = 0.0436. cIMT carotid intima media thickness, CCA common carotid artery, CAA-RI common carotid artery resistance index, CAA-PI common carotid artery pulsatility index, cfPWV carotid-femoral pulse wave velocity, DMARD Disease-modifying anti-rheumatic drugs

In the unadjusted analysis, cfPWV was significantly elevated in the GCA patient group compared to controls [9.90 (8.62–10.80) m/s, vs. 7.57 (6.89–8.16) m/s; *p* < 0.001] (Table 1; Fig. 1C). This difference remained statistically significant even after statistical adjustment for possible confounders like age, sex, BMI, nicotine use, diabetes, and hypertension [cfPWV: 6.203, 95%CI: 2.110-18.235; p_adj_<0.001] (table S6). Even when MAP was added as an additional potential confounder to the above covariates, cfPWV of GCA patients remained significantly higher [cfPWV: 5.539, 95%CI: 1.888–16.246; p_adj_ = 0.002] (Table S7).

Similarly, cIMT was significantly higher in the patient group, both in the unadjusted analysis [0.99 mm (0.90–1.10) vs. 0.83 mm (0.76–0.93); *p* < 0.001] (Table 2; Fig. 1A) and in the adjusted analysis for the six confounders (age, sex, BMI, smoking, diabetes and hypertension) [4.833, 95% CI: 1.101–21.222; *p* < 0.037] (Table S1).

CDUS markers like pulsatility- [CCA-PI 1.92 (1.58–2.22) vs. 1.57 (1.34–1.73); *p* < 0.001, ICA-PI 1.30 (1.10–1.61) vs. (1.18 (0.94–1.42); *p* = 0.019] (Fig. 1B) and resistance indices [CCA-RI 0.79 (0.75–0.83) vs. 0.73 (0.69–0.76); *p* < 0.001, ICA-RI 0.70 (0.65–0.76) vs. 0.66 (0.57–0.73), *p* = 0.010], as well as the plaque area [0.16 (0.07–0.37) cm^2^, vs. 0.045 (0–0.1) cm^2^; *p* < 0.001] were significantly higher compared to controls in unadjusted analyses (Table 2). After adjusting for the same six possible confounders mentioned above, differences of the CCA-PI and CCA-RI remained significant with patients having higher values than controls [CCA-PI: 4.491, 95%CI: 1.458–13.83; p_adj_ =0.009, CCA-RI: 5.153, 95%CI: 1.303–20.379; p_adj_ = 0.019] (Tables S2, S3). However, ICA indices did not retain statistical significance after statistical adjustment [ICA-PI: 2.118, 95%CI: 0.798–5.621; p_adj_ =0.132, ICA-RI: 2.391, 95%CI: 0.872–6.556; p_adj_ =0.090] (Tables S4, S5). 

### Propensity score matching

PSM was used to create a 1:1 matching sample of GCA patients and controls based on age, sex, BMI, diabetes, hypertension, and nicotine use. This matching generated a total of 132 subjects (66 patients and 66 controls) from the above-mentioned initial cohort. The performed standardized mean difference (SMD) analysis shows a good overall balance in the important covariates between the patient and control groups. Overall, the post-matching SMDs are mostly below commonly used thresholds (≈ 0.1–0.5), suggesting that the matching procedure was effective in achieving comparable groups (table S13). The PSM assessment showed statistically significantly higher cfPWV (*p* < 0.001), cIMT (*p* < 0.001), plaques (*p* < 0.001), CCA-RI (*p* < 0.001), CCA-PI (*p* < 0.001) in patients than controls. Consistent with the regression analyses, ICA-PI (*p* = 0.302) and -RI (*p* = 0.092) were not significantly different between the matched subgroups. Moreover, a higher prevalence of subclinical atheromatosis was observed in patients compared with controls (73.9% vs. 55.6%) however, this difference did not reach statistical significance (*p* = 0.107).

### Associations of cfPWV with patient group and disease characteristics

cfPWV showed a moderate correlation with age (*r* = 0.330, *p* = 0.009), cholesterol (*r* = 0.318, *p* = 0.017), and a weak correlation with MAP (rho = 0.257, *p* = 0.044) (Table 3). Moreover, cfPWV associated significantly with CCA-Doppler indices: CCA-PI (rho = 0.402, *p* = 0.012) and CCA-RI (rho = 0.462, *p* = 0.003) (Table 3). Patients under antihypertensive therapy had significantly higher cfPWV values compared to their counterparts [10.5 (8.8–11.2) vs. 9.0 (7.9–10.1), *p* = 0.008] (Table 3). Additionally, in this group, 50 patients (80.6%) were receiving glucocorticoid therapy, with a median current prednisolone dosage of 7.25 mg (IQ 2.88–23.75 mg). 31 patients (50%) were under DMARD therapy (Table 1). The patients not receiving DMARD therapy had significantly higher cfPWV than those on DMARD therapy [10.2 (8.9–11.6) vs. 9.0 (7.9–10.4), *p* = 0.034] (Table 3). The median disease duration in this group was 10 months (IQ 5–33 months) (Table 1).

**Table 3:** Associations between cfPWV, cIMT, and patient characteristics

	cfPWV		cIMT	
	rho/*r*	*p*	rho/*r*	*p*
Age	0.330	< 0.009**	0.355	0.014*
MAP	0.257	0.044*	0.123	0.417
GFR	-0.080	0.567	-0.064	0.699
Creatinine	0.042	0.750	-0.018	0.908
ESR	0.154	0.272	-0.201	0.219
BMI	0.060	0.641	-0.060	0.687
HDL	0.169	0.382	0.058	0.801
LDL	0.240	0.179	-0.419	0.037*
Cholesterol	0.318	0.017*	-0.028	0.861
Heart rate	-0.184	0.152	-0.003	0.987
CRP *(mg/L)*	0.051	0.714	-0.322	0.045
Plaque area *(cm*^*2*^*)*	-	-	0.305	0.047*
CCA-PI	-	-	0.136	0.392
CCA-RI	-	-	0.130	0.412
ICA-PI	-	-	0.166	0.292
ICA-RI	-	-	0.137	0.388
	Median (IQR)	*p*	Median (IQR)	*p*
Gender		0.742		0.864
* Female*	9.9 (8.5–10.8)		0.99 (0.9–1.1)	
* Male*	9.8 (8.9–10.7)		0.96 (0.89–1.38)	
Nicotine use		0.003**		0.062
* Smoker*	8.1 (7.1–9.5)		0.92 (0.87–0.99)	
* Non-smoker*	10.1 (8.9–10.9)		1.1 (0.92–1.2)	
Hypertension		0.058		0.695
* Yes*	10.2 (8.7–10.8)		0.99 (0.91–1.1)	
* No*	9.0 (7.9–9.9)		0.94 (0.88–1.1)	
Statins		0.303		0.446
* Yes*	9.3 (8.3–11.0)		1.05 (0.92–1.25)	
* No*	10.1 (9.1–10.8)		0.99 (0.9–1.1)	
DMARDS		0.034*		0.564
* Yes*	9.0 (7.9–10.4)		1.05 (0.95–1.1)	
* No*	10.2 (8.9–11.6)		0.98 (0.9–1.1)	
Diabetes		0.503		0.569
* Yes*	10.5 (10.5–13.1)		1.0 (0.98–1.33)	
* No*	9.9 (8.6–10.8)		0.99 (0.9–1.1)	
Headache		0.260		0.697
* Yes*	9.9 (8.6–10.6)		0.99 (0.93–1.1)	
* No*	9.9 (8.5–12.1)		1.0 (0.88–1.3)	
Antihypertensive drugs		0.008**		0.268
* Yes*	10.5 (8.8–11.2)		1.0 (0.93–1.1)	
* No*	9.0 (7.9–10.1)		0.93 (0.88–1.1)	
CRP		0.961		0.134
* Yes*	10.1 (8.9–10.8)		0.94 (0.88–0.94)	
* No*	10.2 (8.7–11.0)		1.1 (0.93–1.23)	
SCA				< 0.001***
* Yes*	-	-	1.10 (0.99–1.22)	
* No*	-	-	0.88 (0.82–0.90)	
Plaques				0.234
* Yes*	-	-	1.0 (0.92–1.10)	
* No*	-	-	0.88 (0.84–1.15)	
CV event		0.292		0.812
* Yes*	10.1 (8.8–11.5)		1.05 (0.91–1.10)	
* No*	9.9 (8.3–10.8)		0.99 (0.90–1.10)	
Glucocorticoids		0.569		0.386
Yes	10.1 (8.6–10.8)		1.0 (0.93–1.1)	
No	9.8 (8.4–10.1)		0.9 (0.87–1.6)	
Vision impairment				
Yes	9.8 (8.1–10.8)	0.555	0.99 (0.92–1.1)	0.990
No	10.0 (8.8–10.8)		1.0 (0.89–1.2)	

Quantitative characteristics: Spearman’s (non-normal distribution; rho) and Pearson’s (normal distribution; r) tests. Qualitative characteristics: Mann-Whitney-U test (presentation as median; interquartile-range)

*cfPWV* carotid femoral pulse wave velocity, *MAP* mean arterial pressure, *BMI* body-mass-index, *LDL* low density lipoprotein, *HDL* high density lipoprotein, *GFR* glomerular filtration rate, *CRP* C-reactive protein qualitative, *CCA* common carotid artery, *ICA* internal carotid artery, *PI* pulsatility-index, *RI* resistance-index, *ESR* erythrocyte sedimentation rate, *CV* cardiovascular, *SCA* subclinical carotid atherosclerosis, *DMARDs* disease-modifying anti-rheumatic drugs

* *p* < 0.05

** *p* < 0.01

*** *p* < 0.001

### Associations of carotid sonography parameters with patient group and disease characteristics

**Fig. 2:**
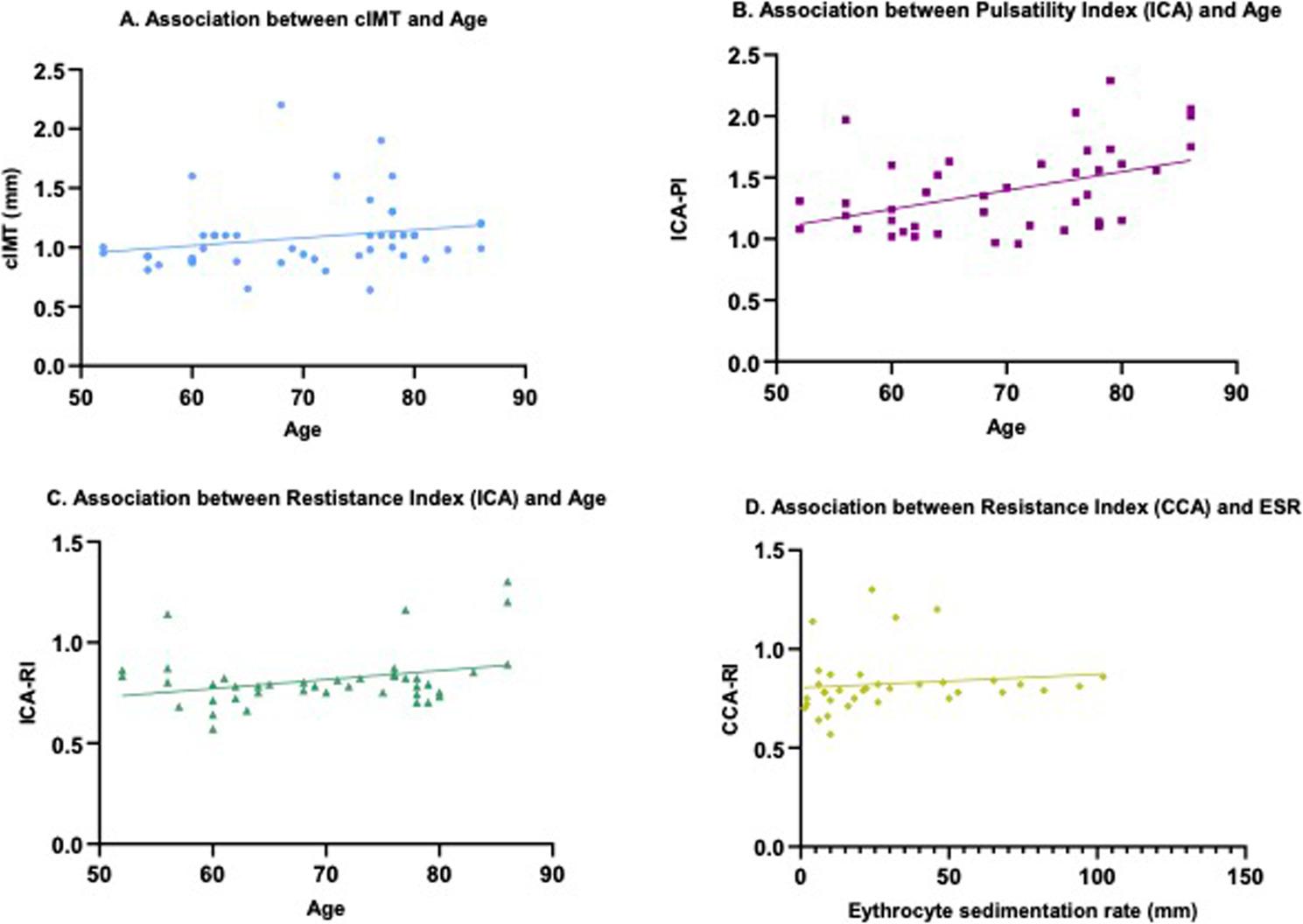
**A**, **B**, **C**, **D**. Association of carotid sonography parameters with patient characteristics. **A**. Association between carotid intima media thickness and age: (*r* = 0.619, *p* <0.001). **B**. Association between pulsatility index and age: (rho = 0.433, *p* = 0.004). **C**. Association between resistance index and age: (rho = 0.414, *p* = 0.006). **D**. Association between resistance index and ESR: (rho = 0.343, *p* = 0.044). cIMT carotid intima media thickness, CCA common carotid artery, ICA internal carotid artery, CAA-RI common carotid artery resistance index, ICA-PI internal carotid artery pulsatility index, ICA-RI internal carotid artery resistance index, ESR erythrocyte sedimentation rate

In the carotid sonography group, cIMT correlated strongly with age (*r* = 0.619, *p* < 0.001) (Fig. 2A) and moderately with the cumulative carotid plaque area (rho = 0.305, *p* = 0.047) (Table 3). Patients with a history of CV events showed bigger plaque area compared to those without such events (0.435 [0.305–0.640] vs. 0.110 [0.040–0.205] cm^2^, *p* < 0.001).ICA-PI and -RI correlated significantly with age (rho = 0.433, *p* = 0.004; rho = 0.414, *p* = 0.006) (Fig. 2B, C) and were higher in patients who were treated with antihypertensive drugs [1.38 (1.15–1.72) vs. 1.12 (1.07–1.42); 0.71 (0.66–0.78) vs. 0.65 (0.63–0.71), all; *p* < 0.05] (Table 4). Moreover, CCA-RI additionally correlated moderately with ESR (rho = 0.343, *p* = 0.044) (Fig. 2D). 35 patients (76.1%) were currently receiving prednisolone therapy, with a median dose of 5 mg (IQ 1.36–16.88), and 20 patients (42.5%) were under DMARDs (Table 2). Both CCA-PI and RI were lower in patients receiving DMARD-therapy [1.5 (1.33–2.04) vs. 1.95 (1.72–2.29); 0.74 (0.68–0.82) vs. 0.80 (0.76–0.85), all; *p* < 0.05] (Table 4; Fig. 1D and E). Patients on prednisolone therapy had higher CCA-PI than patients without (1.99 (1.65–2.36) vs. 1.82 (1.29–1.96); *p* = 0.049) (Table 4). The median disease duration was 11 months (IQ 5–33) (Table 2).

**Table 4:** Associations between CCA (RI, PI) and ICA (RI, PI) and patient characteristics

	CCA				ICA			
	PI		RI		PI		RI	
	rho/*r*	*p*	rho/*r*	*p*	rho/*r*	*p*	rho/*r*^¶^	*p*
Age	0.129	0.416	0.175	0.267	0.433	0.004**	0.414	0.006**
MAP	0.025	0.877	0.036	0.824	-0.179	0.262	-0.193	0.226
GFR	-0.108	0.537	-0.102	0.561	-0.037	0.832	0.040^¶^	0.820
Creatinine	0.144	0.374	0.140	0.561	0.037	0.821	0.019	0.907
ESR	0.186	0.286	0.343	0.044*	0.032	0.857	0.050	0.777
BMI	0.067	0.675	0.343	0.044	0.053	0.739	0.011	0.946
HDL	-0.097	0.691	-0.039	0.875	0.077	0.753	0.258^¶^	0.286
LDL	0.022	0.922	0.071	0.754	-0.144	0.524	-0.036^¶^	0.874
Cholesterol	0.151	0.364	0.121	0.468	-0.088	0.599	-0.058^¶^	0.730
Heart rate	0.030	0.859	0.007	0.968	-0.293	0.074	-0.242^¶^	0.143
CRP *(mg/L)*	-0.020	0.907	0.143	0.414	-0.041	0.815	0.031	0.860
Plaque area *(cm*^*2*^*)*	-0.155	0.346	-0.046	0.781	0.044	0.791	-0.040	0.808
cfPWV	0.402	0.012*	0.462	0.003 **	0.407	0.011*	0.435	0.006**
CCA PI	-	-	0.956	< 0.001***	0.398	0.009 **	0.355	0.021*
CCA RI	0.956	< 0.001***	-	-	0.406	0.008 **	0.377	0.014*
ICA PI	0.398	0.009*	0.406	0.008 **	-	-	0.953	< 0.001***
ICA RI	0.355	0.021*	0.377	0.014*	0.953	< 0.001***	-	-
	Median (IQR)	*p*	Median (IQR)	*p*	Median) (IQR)	*p*	Median (IQR)	*p*
Gender		0.544		0.761		0.748		0.973
* Female*	1.91 (1.6–2.16)		0.79 (0.74–0.83)		1.3 (1.1–1.61)		0.69 (0.65–0.77)	
* Male*	2.16 (1.48–2.38)		0.79 (0.75–0.87)		1.54 (1.1–1.6)		0.71 (0.63–0.76)	
Nicotine		0.337		0.490		0.016*		0.038*
* Smoker*	1.81 (1.43–2.14)		0.79 (0.71–0.82)		1.15 (1.02–1.28)		0.67 (0.92 − 0.70)	
* Non-smoker*	1.94 (1.63–2.29)		0.79 (0.75–0.84)		1.40 (1.11–1.65)		0.71 (0.65–0.78)	
Hypertension		0.219		0.112		0.587		0.647
* Yes*	1.97 (1.64–2.29)		0.8 (0.75–0.85)		1.31 (1.1–1.63)		0.71(0.64–0.78)	
* No*	1.82 (1.4–2.12)		0.76 (0.70–0.79)		1.24 (1.08–1.52)		0.69 (0.65–0.72)	
Statins		0.893		1.00		0.199		0.158
* Yes*	1.92 (1.55–2.36)		0.79 (0.74–0.86)		1.45 (1.23–1.73)		0.72 (0.69–0.80)	
* No*	1.94 (1.66–2.15)		0.8 (0.76–0.84)		1.3 (1.1–1.61)		0.69 (0.65–0.76)	
DMARDs		0.041*		0.028*		0.753		0.688
* Yes*	1.5 (1.33–2.04)		0.74 (0.68–0.82)		1.15 (1.11–1.61)		0.68 (0.64–0.78)	
* No*	1.95 (1.72–2.29)		0.80 (0.76–0.85)		1.35 (1.1–1.61)		0.71 (0.65–0.76)	
Diabetes		0.511		0.158		0.200		0.261
* Yes*	1.97 (1.85–2.19)		0.83 (0.78–0.85)		1.55 (1.30–1.60)		0.74 (0.70–0.76)	
* No*	1.9 (1.48–2.31)		0.78 (0.73–0.82)		1.23 (1.08–1.64)		0.69 (0.64–0.77)	
Headache		0.621		0.472		0.570		0.569
* Yes*	1.91 (1.61–2.33)		0.78 (0.75–0.82)		1.31 (1.06–1.69)		0.69 (0.62–0.78)	
* No*	1.94 (1.55–2.25)		0.80 (0.74–0.86)		1.33 (1.15–1.60)		0.71 (0.65–0.76)	
Antihypertensive drugs		0.248		0.124		0.047*		0.049*
* Yes*	1.97 (1.68–2.26)		0.80 (0.75–0.85)		1.38 (1.15–1.72)		0.71 (0.66–0.78)	
* No*	1.82 (1.42–2.16)		0.76 (0.71–0.82)		1.12 (1.07–1.42)		0.65 (0.63–0.71)	
CRP		1.00		0.477		0.564		0.801
* Yes*	1.93 (1.69–2.12)		0.80 (0.76–0.82)		1.30 (1.07–1.61)		0.71 (0.64–0.76)	
* No*	1.87 (1.49–2.44)		0.78 (0.74–0.87)		1.37 (1.13–1.73)		0.70 (0.65–0.78)	
SCA								
* Yes*	2.0 (1.65–2.34)	0.063	0.81 (0.75–0.86)	0.080	1.37 (1.13–1.69)	0.021*	0.71 (0.65–0.78)	0.070
* No*	1.85 (1.41–1.94)		0.78 (0.70–0.80)		1.11 (1.05–1.27)		0.66 (0.63–0.70)	
Plaques		0.965		0.628		0.660		0.538
* Yes*	1.91 (1.55–2.21)		0.79 (0.75–0.84)		1.30 (1.09–1.61)		0.71 (0.64–0.77)	
* No*	1.95 (1.51–2.16)		0.78 (0.72–0.82)		1.22 (1.11–1.47)		0.68 (0.66–0.69)	
CV event		0.568		0.486		0.956		0.530
* Yes*	2.0 (1.72–2.24)		0.81 (0.76–0.83)		1.44 (1.08–1.56)		0.70 (0.63–0.76)	
* No*	1.92 (1.45–2.22)		0.79 (0.72–0.85)		1.30 (1.11–1.64)		0.70 (0.65–0.78)	
Glucocorticoid		0.049*		0.091		0.549		0.670
* Yes*	1.99 (1.65–2.36)		0.80 (0.75–0.86)		1.31 (1.10–1.63)		0.71 (0.64–0.77)	
* No*	1.82 (1.29–1.96)		0.76 (0.66–0.82)		1.24 (1.11–1.54)		0.69 (0.65–0.74)	
Vision impairment		0.383		0.295		0.699		0.785
* Yes*	1.71 (1.33–2.39)		0.78 (0.7–0.83)		1.38 (1.04–1.97)		0.70 (0.61–0.80)	
* No*	1.95 (1.68–2.20)		0.79 (0.75–0.84)		1.30 (1.11–1.56)		0.70 (0.65–0.76)	

Quantitative characteristics: Spearman’s (non-normal distribution; rho) and Pearson’s ^¶^ (normal distribution; r) tests

Qualitative characteristics: Mann-Whitney-U test ( presentation as median; interquartile-range**)**

*cfPWV* carotid femoral pulse wave velocity, *CCA* common carotid artery, *ICA* internal carotid artery, *RI* resistance-index, *PI* pulsatility-index, *ESR* erythrocyte sedimentation rate, *MAP* mean arterial pressure, *BMI* body-mass-index, *LDL* low density lipoprotein, *HDL* high density lipoprotein, *GFR* glomerular filtration rate, *CRP* C-reactive protein qualitative, *ESR* erythrocyte sedimentation rate, qual qualitative, *Cv* cardiovascular, *SCA* subclinical carotid atherosclerosis, *DMARDs* disease-modifying anti-rheumatic drugs

* *p* < 0.05

** *p* < 0.01

*** *p* < 0.001

### Definition of disease status and details of therapy

43% of the patients had active disease according to the laboratory parameters at the time of the examination, and 57% showed signs of active GCA on imaging (MRI or US). 18 of active patients (42%) had both imaging and laboratory activity signs, and 13 patients showed no activity in either imaging or laboratory tests. At the time of assessment, 31 patients were receiving DMARDs, and 30 patients were treated with glucocorticoids (GC) but not with DMARDs. In total, 56 patients (85%) were under current GC therapy. In total, six different DMARD agents were used: Tocilizumab (*n* = 16), Methotrexate (*n* = 11), Azathioprine (*n* = 2), Leflunomide (*n* = 1), a TNF-α blocker (Etanercept; *n* = 1), and a JAK inhibitor (Baricitinib; *n* = 1) (Tables 1 and 2).

### Extracranial GCA vs. cranial-only disease vs. overlap extracranial/cranial: associations of cfPWV, Doppler parameters, and plaque are with different phenotypes

The proportion of extracranial GCA versus cranial-only disease or their overlap was as follows: Cranial involvement was present in 43 patients (64%), extracranial manifestations in 17 patients (26%), and an overlap of cranial and extracranial GCA in 7 patients (11%), resulting in a total of 66 patients included in the analysis (Table 5).

**Table 5:** Surrogate cardiovascular markers in patients with extracranial GCA, cranial-only GCA, and overlap extracranial/cranial disease

	extracranial	cranial	overlap
Patient count (*n*; %)	17 (25.8%)	42 (63.6%)	7 (10.6%)
cfPWV (m/s)	9.92 ± (2.41)	9,89 (8.88–10.64)	10.11± (1.88)
Plaque area (mm^2^)	0.32 ± (0.38)	0.19 (0.07–0.39)	0.35 ± (0.26)
CCA PI	2.13 ± (0.73)	1.85 (1.58–2.01)	2.59 ± (1.196)
CCA RI	0.81 ± (0.14)	0.78 (0.75–0.81)	0.92 ± (0.27)
ICA PI	1.39 ± (0.295)	1.23 (1.07–1.56)	1.87 ± (0.33)
ICA RI	0.70 ± (0.63)	0.69 ± (0.06)	0.78 ± (0.07)

All non-normal distributions: presentation as median (interquartile range)

t-test (normal distribution; mean ± SD)

Mann-Whitney-U test ( non-normal distribution; median, interquartile-range)

*cfPWV* carotid femoral pulse wave velocity, *CCA* common carotid artery, *ICA* internal carotid artery, *RI* resistance index, *PI* pulsatility index

*normal distribution: presentation as mean ± standard deviation*

Data on the proportion of extracranial GCA vs. cranial-only GCA vs. overlap extracranial/cranial disease are seen in Table 5. To examine potential differences between the different phenotypes, we performed several regression analyses adjusting for disease duration and current glucocorticoid dose at the time of examination. In these analyses, no significant differences in cfPWV and CCA-RI were observed between the cranial, extracranial, or overlap subtypes (all *p* > 0.05) (Table S8, S12). Similarly, no significant differences were found between extracranial and cranial for ICA-PI and -RI, CCA-PI, and plaque area (all *p* > 0.05) (Table S9-S11).

On the other hand, statistically, significant higher values were observed for overlap compared to the cranial phenotypes for CCA-PI [ 2.59 ± (1,196) vs. 1.88 ± (0.48)*p* = 0.033], ICA-RI [ 0.78 ± (0.07) vs. 0.69 ± (0.06) *p* = 0.011]and -PI [ 1.87 ± (0.33) vs. 1.32 ± (0.32)*p* = 0.002] and increased results for overlap compared to the extracranial phenotypes for plaque area [ 0.35 ± (0.26) vs. 0.32 ± (0.38) *p* = 0.005], ICA-PI [1.87 ± (0.33) vs. 1.39 ± (0.295) *p* = 0.030] (Table S9-S11).

## Discussion

In this study, we showed that patients with GCA had higher carotid resistance, pulsatility, and aortic stiffness compared to controls. These differences remained significant even after statistical adjustment and PSM analyses for several well-established confounding factors. Moreover, we found a higher carotid calcification burden and an inverse association between CV/cerebrovascular surrogates and the presence of DMARD medication in GCA patients. Importantly, patient- and disease-associated predictors of high CV/CVB risk were also established.

To our knowledge, this study is the first to assess this wide combination of CV and CVB surrogate parameters in a large cohort of patients with GCA. Moreover, it is the first study to show a possible benefit of immunosuppressive treatment on both aortic stiffness and carotid compliance as assessed by CDUS in the context of this rare condition.

Previous studies on carotid sonography in GCA have primarily focused on greyscale parameters of arterial structure, such as cIMT, particularly in the context of vasculitis involvement. However, quantitative data on vascular calcification are limited, despite its potential to better assess CV risk by reflecting plaque burden and its link to stenosis and adverse outcomes [30]. Importantly, the predictive value of cIMT has been increasingly criticized during the last years, since increases in IMT do not seem to be an accurate marker of CVB or CV risk [31].

Both examined CDUS markers, RI and PI, have been shown to correlate with CV events in the general population [15, 32]. In particular, increased carotid flow pulsatility has been associated with a higher risk of stroke, while abnormal RI reflects higher arterial resistance associating with atherosclerosis [15]. Elevated carotid PI reflects greater force exerted on cerebral small vessels, potentially leading to vessel damage, small infarctions, and hemorrhages [33]. Since patients with GCA have an increased risk of stroke and myocardial infarction, the here presented Doppler parameters could serve as a valuable method for risk stratification [34, 35].

cfPWV was similarly significantly higher in patients, suggesting increased aortic stiffness compared to controls. Importantly, aortic stiffness is an independent predictor of CV and overall mortality in the general population, and cfPWV can provide valuable information about the vascular compliance of the aorta [12, 36, 37]. This is clinically relevant, as increased aortic stiffness has significant hemodynamic consequences. Reduced vascular compliance impairs the ability of arteries to buffer pulsatile pressure changes, leading to elevated systolic arterial pressure and widened pulse pressure. These changes can increase the mechanical load on the heart and promote microvascular damage in target organs [36]. Patients with increased aortic stiffness have been shown to have a higher risk of stroke, heart failure, and coronary artery disease [36, 37].

The number of studies that simultaneously analyzed cfPWV and cIMT in GCA patients is low [38, 39]. Emamifar et al. [38] followed 77 patients over 40 weeks to assess the effects of inflammation and glucocorticoids on aortic stiffness. Treatment reduced arterial stiffness, likely due to decreased inflammation. Aortic PWV correlated with age, male sex, arterial pressure, and CRP levels. Baseline PWV was higher in PMR and GCA patients compared to matched controls. In a 2012 study, Jod et al. assessed aortic dilatation (AD) in 144 GCA patients and 115 controls using aortic CT scans, adjusting for age, sex, and body surface area [39]. While PWV and IMT showed no significant group differences, multivariate analysis linked PMR to increased cIMT and thoracic AD.

Additional studies on imaging markers in GCA have mainly examined inflammatory wall thickening and vascular damage (e.g., aneurysms, dissections) using grey-scale ultrasound and PET-CT [40–42]. While essential for GCA monitoring, the lack of clear remission definitions complicates treatment [40].

### Associations of surrogate markers and patient characteristics

In our cohort, we found statistically significantly lower values of cfPWV and Doppler Indices (CCA) in patients under DMARD therapy than those without. Therefore, a CV-protective effect of DMARDs, possibly via reduction of the cumulative inflammatory activity, can be discussed, although this relationship should be seen as associative. Interestingly, different studies have examined the impact of immunosuppressants on CV surrogates in autoimmune rheumatic diseases, particularly focusing on PWV in RA. For example, Provan et al. longitudinally studied 36 RA patients treated with tocilizumab, rituximab, or abatacept. Tocilizumab (*n* = 7) led to reduced PWV, CRP, ESR, and BP after 3 months (*p* = 0.03). Rituximab (*n* = 24) also significantly reduced PWV after 12 months [43]. On the contrary, McInnes et al. were not able to find an effect of tocilizumab on PWV in 132 patients with RA during a longitudinal randomized controlled trial [44]. The decrease in PWV after 12 weeks was higher with placebo than under tocilizumab (mean difference 0.79 m/s (95%CI 0.22 to 1.35; *p* = 0.0067) [44]. We are not aware of studies having shown beneficial effects on the resistance and compliance of multiple large arteries in GCA.

In addition, we demonstrated a correlation between CCA-RI and ESR. The relationship between impaired arterial health and autoimmune disease-associated inflammation has been extensively discussed, with several potential mechanisms proposed to explain this interaction. Key biological factors contributing to inflammation-induced arterial wall stiffening include a reduced elastin/collagen ratio, oxidative stress (triggered by reactive oxygen species, ROS), vascular calcification, and endothelial dysfunction, along with changes in vascular smooth muscle cells [45]. Moreover, accelerated atherosclerosis resulting from systemic inflammation can also contribute to increased arterial stiffness.

Furthermore, both cfPWV and cIMT showed a strong correlation with age. This association has been well-documented in both the healthy population [46, 47] and individuals with rheumatological diseases [10, 17, 19, 20]. These changes reflect age-associated pathophysiological processes such as arterial collagen accumulation, elastin loss, and vessel wall thickening, ultimately leading to increased arterial stiffness.

Additionally, cfPWV correlated with factors such as MAP at the time of measurement and chronic arterial hypertension. It is well-established that elevated blood pressure contributes to increased arterial stiffness by exerting repeated mechanical stress on the vessel walls [48]. Regarding smoking, a known promoter of atherosclerosis [49], direct correlations with both cfPWV and cIMT were shown in our analyses. These findings underscore, cumulatively, the significant role of traditional CV risk factors.

Another intriguing finding of our study concerns the established association between prednisolone therapy and increased CCA-PI. Previous research has demonstrated that arterial stiffness can be reduced under glucocorticoid treatment in this patient population [38]. However, the effects of GC on arterial health are difficult to evaluate. GC can, on the one hand, reduce inflammation, but on the other hand, can nevertheless lead to impaired glucose and lipid metabolism, leading to conditions that may increase CV risk [50].

Die vorliegende Studie wurde als explorative technische und methodische Untersuchung konzipiert, um die Machbarkeit, Reproduzierbarkeit und das diskriminatorische Potenzial ausgewählter nicht-invasiver vaskulärer Indizes bei Patientinnen und Patienten mit Riesenzellarteriitis (GCA) zu untersuchen. Ziel war es dabei ausdrücklich nicht, unmittelbare Konsequenzen für die klinische Routine oder bestehende Nachsorge- und Therapiestrategien abzuleiten.

### Clinical relevance and perspective

As patients with GCA are typically older and more frequently affected by CV comorbidities, an important added value of the present work lies in demonstrating that advanced non-invasive vascular measurements are able to detect disease-associated vascular alterations even after adjustment for age and CV risk factors. Notably, cfPWV and carotid ultrasound parameters have been shown to possess high CV predictive value and are therefore recommended as adjunctive tools for CV risk stratification [51]. To place the vascular measurements into a clinically relevant context, we examined their relationship with established CV disease and key disease-specific characteristics of GCA. Analyses accounted for prior CV events as well as major clinical phenotypes and manifestations of GCA using multivariable adjustment and propensity score matching to minimize confounding.From a clinical perspective, the results should be interpreted as hypothesis-generating rather than as a basis for immediate therapeutic decisions. Nevertheless, the findings suggest that selected vascular parameters may capture aspects of vascular involvement in GCA that extend beyond conventional clinical assessment. The study complements existing longitudinal and population-based cohort studies by providing a detailed methodological and functional evaluation of vascular measurements in an expert setting.

### Limitations

Our study has several limitations. First, it does not allow for any prognostic conclusions regarding the value of included CV surrogate markers and their relation to actual mortality or morbidity, as it was performed in a cross-sectional manner. However, both cfPWV and carotid ultrasonography have previously demonstrated high predictive value for CV events, both in the general population and in autoimmune diseases [12–16].

Another potential limitation is the difference in some patient characteristics (e.g. age) between groups. To minimize bias, we applied both regression and propensity score matching analyses for key CV risk factors, like age, sex, BMI, diabetes, nicotine use, and arterial hypertension. Notably, cIMT, cfPWV, CCA-RI, and CCA-PI remained significantly elevated in the GCA group, indicating these findings are largely independent of those confounders. Additionally, the potential effects of immunosuppressants and glucocorticoids on arterial health should be considered. DMARD therapy was associated with lower values for surrogate markers, while prednisolone therapy did not show strong statistical associations with most of the assessed indices, except for CCA-PI. However, only the current, rather than cumulative, prednisolone dose was documented, which represents a limitation. Extracranial GCA tends to occur in patients who are younger, more often female, and who frequently exhibit longer disease duration, greater glucocorticoid exposure, and an overall less favorable CV risk profile. Because DMARD therapy is often initiated in patients with more active, complicated, or metabolically burdened disease, treatment allocation is inherently non-random. As a result, the association observed between DMARD exposure and more favorable vascular indices may partly reflect indication bias in addition to potential treatment effects. Therefore, this finding should be interpreted with caution and ideally confirmed in longitudinal studies that compare vascular parameters before and after DMARD initiation.

In conclusion, our study demonstrates significantly elevated levels of carotid resistance, pulsatility, aortic stiffness, and atherosclerosis in patients with GCA. To the best of our knowledge, this is the first exploration to integrate a combination of CV surrogate markers and angiopathy indicators - including cfPWV, cIMT, vascular Doppler indices, and plaque screening - within a GCA cohort. While the present findings are exploratory, they demonstrate the feasibility and discriminatory potential of advanced non-invasive vascular measurements in an expert setting. In this context, our work complements existing longitudinal and population-based studies by providing a detailed technical and methodological assessment of non-invasive vascular measurements. Innovative surrogate markers, such as carotid PI and RI, could play a crucial role in the staging and diagnosis of GCA, offering a more comprehensive assessment of the large arterial tree. Future longitudinal, multicenter studies are needed to assess the predictive value of these well-established and novel markers.

## Supplementary Information

Supplementary Material 1.

## Data Availability

The data analysed during the current study are available from the corresponding author on reasonable request.

## Abbreviations

AD: Aortic dissection
BMI: Body mass index
CCA: Common carotid artery
CDUS: Color Doppler ultrasound
cfPWV: Carotid–femoral pulse wave velocity
cIMT: Carotid intima media thickness
CRP: C–reactive protein
CT: Computed Tomography
CV: Cardiovascular
CVB: Cerebrovascular
DMARD: Disease–modifying anti–rheumatic drug
EDV: End–diastolic velocity
ESC: European Society of Cardiology
ESR: Erythrocyte sedimentation rate
GCA: Giant cell arteritis
GSUS: Greyscale ultrasound
ICA: Internal carotid artery
MAP: Mean arterial pressure
MRI: Magnetic Resonance Imaging
PET: CT–Positron Emission Tomography und Computed Tomography
PI: Pulsatility index
PSM: Propensity score matching
PSV: Peak systolic velocity
RA: Rheumatoid arthritis
RI: Resistance index
SCA: Subclinical carotid atherosclerosis
SCORE: Systematic Coronary Risk Evaluation
US: Ultrasound
VASCARD: Vasculitis–associated cardiovascular risk
