# Supplementary material for: Color Doppler carotid resistance, pulsatility, and aortic oscillometry indices in giant cell arteritis: insights from the VASCARD cohort
Source: Arthritis Res Ther. 2026 Mar 28;28:84. doi: 10.1186/s13075-026-03779-w (PMC13063443; doi:10.1186/s13075-026-03779-w)
Supplement: Supplementary file 1 — Supplementary Material 1. [file 13075_2026_3779_MOESM1_ESM.docx]

**Supplementary material**

**Table S1.** Binary logistic regression of cIMT

|  | | **B** | **S.E.** | **Wald** | **df** | **Sig.** | **Exp(B)** | **95% C.I.for EXP(B)** | |
| --- | --- | --- | --- | --- | --- | --- | --- | --- | --- |
|  |  |  |  |  |  |  |  | **Lower** | **Upper** |
|  | **cIMT** | 1.575 | .755 | 4.355 | 1 | .037 | 4.833 | 1.101 | 21.222 |
|  | **Age** | .162 | .057 | 8.015 | 1 | .005 | 1.176 | 1.051 | 1.316 |
|  | **BMI** | -.178 | .091 | 3.817 | 1 | .051 | .837 | .700 | 1.001 |
|  | **Hypertension** | .833 | .757 | 1.210 | 1 | .271 | 2.300 | .521 | 10.149 |
|  | **Sex** | .661 | .922 | .514 | 1 | .473 | 1.936 | .318 | 11.790 |
|  | **Nicotine use** | 1.912 | .959 | 3.972 | 1 | .046 | 6.764 | 1.032 | 44.326 |
|  | **Diabetes** | 1.523 | 1.347 | 1.278 | 1 | .258 | 4.585 | .327 | 64.299 |
|  | **Constant** | -6.530 | 4.132 | 2.498 | 1 | .114 | .001 |  |  |

*cIMT:* carotid intima-media thickness, *BMI:* body mass index

**Table S2.** Binary logistic regression of CCA PI

|  | | **B** | **S.E.** | **Wald** | **df** | **Sig.** | **Exp(B)** | **95% C.I.for EXP(B)** | |
| --- | --- | --- | --- | --- | --- | --- | --- | --- | --- |
|  |  |  |  |  |  |  |  | **Lower** | **Upper** |
|  | **CCA PI** | 1.502 | .574 | 6.852 | 1 | .009 | 4.491 | 1.458 | 13.830 |
|  | **Age** | .242 | .073 | 11.118 | 1 | <.001 | 1.274 | 1.105 | 1.468 |
|  | **BMI** | -.223 | .103 | 4.733 | 1 | .030 | .800 | .654 | .978 |
|  | **Hypertension** | .979 | .866 | 1.279 | 1 | .258 | 2.662 | .488 | 14.519 |
|  | **Sex** | 1.253 | 1.064 | 1.387 | 1 | .239 | 3.502 | .435 | 28.210 |
|  | **Nicotine use** | 2.720 | 1.024 | 7.057 | 1 | .008 | 15.179 | 2.040 | 112.912 |
|  | **Diabetes** | .879 | 1.490 | .348 | 1 | .555 | 2.408 | .130 | 44.706 |
|  | **Constant** | -11.081 | 4.869 | 5.179 | 1 | .023 | .000 |  |  |

*CCA PI*: common carotid artery pulsatility index, *BMI*: body mass index

**Table S3.** Binary logistic regression of CCA RI

|  | | **B** | **S.E.** | **Wald** | **df** | **Sig.** | **Exp(B)** | **95% C.I.for EXP(B)** | |
| --- | --- | --- | --- | --- | --- | --- | --- | --- | --- |
|  |  |  |  |  |  |  |  | **Lower** | **Upper** |
|  | **CCA RI** | 1.639 | .702 | 5.461 | 1 | .019 | 5.153 | 1.303 | 20.379 |
|  | **Age** | .210 | .066 | 10.128 | 1 | .001 | 1.234 | 1.084 | 1.405 |
|  | **BMI** | -.234 | .101 | 5.325 | 1 | .021 | .792 | .649 | .965 |
|  | **Hypertension** | .926 | .818 | 1.280 | 1 | .258 | 2.523 | .508 | 12.545 |
|  | **Sex** | 1.047 | 1.043 | 1.008 | 1 | .315 | 2.849 | .369 | 21.995 |
|  | **Nicotine use** | 2.444 | .984 | 6.170 | 1 | .013 | 11.522 | 1.675 | 79.280 |
|  | **Diabetes** | .910 | 1.472 | .383 | 1 | .536 | 2.486 | .139 | 44.460 |
|  | **Constant** | -8.570 | 4.617 | 3.446 | 1 | .063 | .000 |  |  |

*CCA RI*: common carotid artery resistance index, *BMI*: body mass index

**Table S4.** Binary logistic regression of ICA PI

|  | | **B** | **S.E.** | **Wald** | **df** | **Sig.** | **Exp(B)** | **95% C.I.for EXP(B)** | |
| --- | --- | --- | --- | --- | --- | --- | --- | --- | --- |
|  |  |  |  |  |  |  |  | **Lower** | **Upper** |
|  | **ICA PI** | .751 | .498 | 2.272 | 1 | .132 | 2.118 | .798 | 5.621 |
|  | **Age** | .241 | .077 | 9.792 | 1 | .002 | 1.272 | 1.094 | 1.480 |
|  | **BMI** | -.252 | .096 | 6.856 | 1 | .009 | .777 | .644 | .939 |
|  | **Hypertension** | .517 | .782 | .437 | 1 | .508 | 1.677 | .362 | 7.766 |
|  | **Sex** | 1.840 | 1.191 | 2.385 | 1 | .122 | 6.296 | .610 | 65.020 |
|  | **Nicotine use** | 2.431 | 1.006 | 5.839 | 1 | .016 | 11.367 | 1.583 | 81.636 |
|  | **Diabetes** | 1.467 | 1.413 | 1.078 | 1 | .299 | 4.336 | .272 | 69.142 |
|  | **Constant** | -10.478 | 4.938 | 4.502 | 1 | .034 | .000 |  |  |

*ICA PI:* internal carotid artery pulsatility index, *BMI:* body mass index

**Table S5.** Binary logistic regression of ICA RI

|  | | **B** | **S.E.** | **Wald** | **df** | **Sig.** | **Exp(B)** | **95% C.I.for EXP(B)** | |
| --- | --- | --- | --- | --- | --- | --- | --- | --- | --- |
|  |  |  |  |  |  |  |  | **Lower** | **Upper** |
|  | **ICA RI** | .872 | .515 | 2.867 | 1 | .090 | 2.391 | .872 | 6.556 |
|  | **Age** | .228 | .075 | 9.264 | 1 | .002 | 1.256 | 1.085 | 1.455 |
|  | **BMI** | -.229 | .095 | 5.845 | 1 | .016 | .796 | .661 | .958 |
|  | **Hypertension** | .454 | .798 | .324 | 1 | .569 | 1.574 | .330 | 7.516 |
|  | **Sex** | 1.547 | 1.163 | 1.771 | 1 | .183 | 4.699 | .481 | 45.889 |
|  | **Nicotine use** | 2.110 | .991 | 4.534 | 1 | .033 | 8.245 | 1.183 | 57.471 |
|  | **Diabetes** | 1.234 | 1.431 | .743 | 1 | .389 | 3.433 | .208 | 56.681 |
|  | **Constant** | -9.930 | 4.903 | 4.102 | 1 | .043 | .000 |  |  |

*ICA RI:* internal carotid artery resistance index, *BMI:* body mass index

**Table S6.** Binary logistic regression of cfPWV

|  | | **B** | **S.E.** | **Wald** | **df** | **Sig.** | **Exp(B)** | **95% C.I.for EXP(B)** | |
| --- | --- | --- | --- | --- | --- | --- | --- | --- | --- |
|  |  |  |  |  |  |  |  | **Lower** | **Upper** |
|  | **cfPWV** | 1.825 | .550 | 11.003 | 1 | <.001 | 6.203 | 2.110 | 18.235 |
|  | **Age** | .153 | .050 | 9.261 | 1 | .002 | 1.166 | 1.056 | 1.286 |
|  | **BMI** | -.139 | .072 | 3.729 | 1 | .053 | .871 | .756 | 1.002 |
|  | **Hypertension** | 1.081 | .617 | 3.071 | 1 | .080 | 2.949 | .880 | 9.882 |
|  | **Sex** | .238 | .777 | .094 | 1 | .760 | 1.268 | .277 | 5.813 |
|  | **Nicotine use** | 1.908 | .767 | 6.197 | 1 | .013 | 6.743 | 1.501 | 30.298 |
|  | **Diabetes** | 2.221 | 1.423 | 2.436 | 1 | .119 | 9.219 | .567 | 150.020 |
|  | **Constant** | -7.452 | 3.840 | 3.766 | 1 | .052 | .001 |  |  |

*cfPWV*: carotid-femoral pulse wave velocity, *BMI:* body mass index

**Table S7.** Binary logistic regression of cfPWV with MAP

|  | | **B** | **S.E.** | **Wald** | **df** | **Sig.** | **Exp(B)** | **95% C.I.for EXP(B)** | |
| --- | --- | --- | --- | --- | --- | --- | --- | --- | --- |
|  |  |  |  |  |  |  |  | **Lower** | **Upper** |
|  | **cfPWV** | 1.712 | .549 | 9.721 | 1 | .002 | 5.539 | 1.888 | 16.246 |
|  | **Age** | .161 | .052 | 9.721 | 1 | .002 | 1.174 | 1.062 | 1.299 |
|  | **BMI** | -.139 | .071 | 3.852 | 1 | .050 | .871 | .758 | 1.000 |
|  | **Hypertension** | 1.011 | .622 | 2.645 | 1 | .104 | 2.750 | .813 | 9.304 |
|  | **Sex** | .372 | .807 | .213 | 1 | .645 | 1.451 | .298 | 7.053 |
|  | **Nicotine use** | 1.758 | .783 | 5.033 | 1 | .025 | 5.798 | 1.249 | 26.923 |
|  | **Diabetes** | 2.353 | 1.417 | 2.758 | 1 | .097 | 10.520 | .654 | 169.166 |
|  | **MAP** | .024 | .026 | .804 | 1 | .370 | 1.024 | .972 | 1.078 |
|  | **Constant** | -10.307 | 5.036 | 4.189 | 1 | .041 | .000 |  |  |

*MAP*: mean arterial pressure, *cfPWV*: carotid-femoral pulse wave velocity, *BMI:* body mass index

**Tables S8.** Regression analyses of cfPWV adjusted for phenotype, disease duration, and glucocorticoid dose.

| **Model** | | **Unstandardized Coefficients** | | **Standardized Coefficients** | **t** | **Sig.** |
| --- | --- | --- | --- | --- | --- | --- |
|  |  | **B** | **Std. Error** | **Beta** |  |  |
|  | **(Constant)** | 9.884 | .997 |  | 9.913 | <.001 |
|  | **cranial/extracranial** | -.031 | .631 | -.007 | -.049 | .961 |
|  | **disease duration** | .005 | .009 | .084 | .563 | .576 |
|  | **current prednisolone dosage** | -.001 | .007 | -.017 | -.108 | .914 |
|  | a. Dependent Variable: cfPWV | | | | | |

| **Model** | | **Unstandardized Coefficients** | | **Standardized Coefficients** | **t** | **Sig.** |
| --- | --- | --- | --- | --- | --- | --- |
|  |  | **B** | **Std. Error** | **Beta** |  |  |
|  | **(Constant)** | 9.788 | 1.063 |  | 9.212 | <.001 |
|  | **cranial/overlap** | .146 | .786 | .029 | .185 | .854 |
|  | **disease duration** | .002 | .009 | .033 | .201 | .842 |
|  | **current prednisolone dosage** | -.001 | .007 | -.016 | -.098 | .922 |
| a. Dependent Variable: cfPWV | | | | | | |

| **Model** | | **Unstandardized Coefficients** | | **Standardized Coefficients** | **t** | **Sig.** |
| --- | --- | --- | --- | --- | --- | --- |
|  |  | **B** | **Std. Error** | **Beta** |  |  |
|  | **(Constant)** | 9.134 | 1.408 |  | 6.487 | <.001 |
|  | **extracranial/overlap** | .128 | .937 | .029 | .137 | .893 |
|  | **disease duration** | .043 | .025 | .382 | 1.741 | .098 |
|  | **current prednisolone dosage** | -.024 | .029 | -.179 | -.848 | .407 |
| a. Dependent Variable: cfPWV | | | | | | |

cfPWV: carotid-femoral pulse wave velocity

**Tables S9.** Regression analyses of Plaque area adjusted for phenotype, disease duration, and glucocorticoid dose

| **Model** | | | | **Unstandardized Coefficients** | | | | **Standardized Coefficients** | **t** | | **Sig.** | |
| --- | --- | --- | --- | --- | --- | --- | --- | --- | --- | --- | --- | --- |
|  |  |  |  | **B** | | **Std. Error** | | **Beta** |  |  |  |  |
| 1 | | **(Constant)** | | .532 | | .178 | |  | 2.989 | | .005 | |
|  |  | **cranial/extracranial** | | -.177 | | .117 | | -.246 | -1.513 | | .139 | |
|  |  | **disease duration** | | -.001 | | .002 | | -.086 | -.510 | | .613 | |
|  |  | **current prednisolone dosage** | | .000 | | .001 | | -.054 | -.317 | | .753 | |
|  | | a. Dependent Variable: Plaque area | | | | | | | | | | |
|  | | | | | | | | | | | | |
| **Model** | | | **Unstandardized Coefficients** | | | | **Standardized Coefficients** | | | **t** | | **Sig.** |
|  |  |  | **B** | | **Std. Error** | | **Beta** | | |  |  |  |
| 1 | **(Constant)** | | .376 | | .289 | |  | | | 1.301 | | .204 |
|  | **cranial/overlap** | | .004 | | .236 | | .004 | | | .019 | | .985 |
|  | **disease duration** | | -.002 | | .003 | | -.147 | | | -.730 | | .472 |
|  | **current prednisolone dosage** | | -.001 | | .002 | | -.083 | | | -.412 | | .684 |
| a. Dependent Variable: Plaque area | | | | | | | | | | | | |

|  | | | | | | |
| --- | --- | --- | --- | --- | --- | --- |
| **Model** | | **Unstandardized Coefficients** | | **Standardized Coefficients** | **t** | **Sig.** |
|  |  | **B** | **Std. Error** | **Beta** |  |  |
| 1 | **(Constant)** | -.098 | .108 |  | -.912 | .381 |
|  | **extracranial/overlap** | .254 | .074 | .667 | 3.446 | .005 |
|  | **disease duration** | .000 | .002 | .048 | .231 | .821 |
|  | **current prednisolone dosage** | -.003 | .002 | -.326 | -1.579 | .143 |
| a. Dependent Variable: Plaque area | | | | | | |

**Tables S9.** Regression analyses of ICA PI adjusted for phenotype, disease duration, and glucocorticoid dose.

| **Model** | | Unstandardized Coefficients | | Standardized Coefficients | t | Sig. |
| --- | --- | --- | --- | --- | --- | --- |
|  |  | B | Std. Error | Beta |  |  |
| 1 | **(Constant)** | 1.153 | .169 |  | 6.809 | <.001 |
|  | **cranial/extracranial** | .086 | .109 | .133 | .796 | .431 |
|  | **disease duration** | .003 | .002 | .263 | 1.511 | .140 |
|  | **current prednisolone dosage** | .000 | .001 | .042 | .242 | .811 |
| a. Dependent Variable: ICA PI | | | | | | |

| **Model** | | **Unstandardized Coefficients** | | **Standardized Coefficients** | **t** | **Sig.** |
| --- | --- | --- | --- | --- | --- | --- |
|  |  | **B** | **Std. Error** | **Beta** |  |  |
| **1** | **(Constant)** | .618 | .222 |  | 2.786 | .010 |
|  | **cranial/overlap** | .595 | .171 | .565 | 3.483 | .002 |
|  | **disease duration** | .003 | .002 | .270 | 1.593 | .123 |
|  | **current prednisolone dosage** | .001 | .001 | .116 | .692 | .495 |
| a. Dependent Variable: ICA PI | | | | | | |

| **Model** | | **Unstandardized Coefficients** | | **Standardized Coefficients** | **t** | **Sig.** |
| --- | --- | --- | --- | --- | --- | --- |
|  |  | **B** | **Std. Error** | **Beta** |  |  |
| **1** | **(Constant)** | 1.053 | .271 |  | 3.882 | .002 |
|  | **extracranial/overlap** | .433 | .176 | .573 | 2.459 | .030 |
|  | **disease duration** | -.003 | .004 | -.178 | -.713 | .489 |
|  | **current prednisolone dosage** | -.003 | .004 | -.190 | -.763 | .460 |
| a. Dependent Variable: ICA PI | | | | | | |

*ICA: internal carotid artery, PI: pulsatility-index*

**Tables S10.** Regression analyses of ICA RI adjusted for phenotype, disease duration, and glucocorticoid dose.

| **Model** | | **Unstandardized Coefficients** | | **Standardized Coefficients** | **t** | **Sig.** |
| --- | --- | --- | --- | --- | --- | --- |
|  |  | **B** | **Std. Error** | **Beta** |  |  |
| **1** | **(Constant)** | .666 | .036 |  | 18.611 | <.001 |
|  | **cranial/extracranial** | .015 | .023 | .113 | .663 | .512 |
|  | **disease duration** | .000 | .000 | .133 | .746 | .461 |
|  | **current prednisolone dosage** | .000 | .000 | .085 | .477 | .636 |
| a. Dependent Variable: ICA RI | | | | | | |

| **Model** | | **Unstandardized Coefficients** | | **Standardized Coefficients** | **t** | **Sig.** |
| --- | --- | --- | --- | --- | --- | --- |
|  |  | **B** | **Std. Error** | **Beta** |  |  |
| **1** | **(Constant)** | .575 | .046 |  | 12.517 | <.001 |
|  | **cranial/overlap** | .097 | .035 | .473 | 2.733 | .011 |
|  | **disease duration** | .000 | .000 | .204 | 1.128 | .270 |
|  | **current prednisolone dosage** | .000 | .000 | .207 | 1.153 | .260 |
| a. Dependent Variable: ICA RI | | | | | | |

| **Model** | | **Unstandardized Coefficients** | | **Standardized Coefficients** | **t** | **Sig.** |
| --- | --- | --- | --- | --- | --- | --- |
|  |  | **B** | **Std. Error** | **Beta** |  |  |
| 1 | **(Constant)** | .673 | .054 |  | 12.394 | <.001 |
|  | **extracranial/overlap** | .068 | .035 | .464 | 1.939 | .076 |
|  | **disease duration** | -.001 | .001 | -.355 | -1.383 | .192 |
|  | **current prednisolone dosage** | -.001 | .001 | -.289 | -1.133 | .279 |
| a. Dependent Variable: ICA RI | | | | | | |

*ICA: internal carotid artery , RI: resistance-index*

**Tables S11.** Regression analyses of CCA PI adjusted for phenotype, disease duration, and glucocorticoid dose.

| **Model** | | **Unstandardized Coefficients** | | **Standardized Coefficients** | **t** | **Sig.** |
| --- | --- | --- | --- | --- | --- | --- |
|  |  | **B** | **Std. Error** | **Beta** |  |  |
| **1** | **(Constant)** | 1.423 | .308 |  | 4.626 | <.001 |
|  | **cranial/extracranial** | .293 | .197 | .241 | 1.486 | .147 |
|  | **disease duration** | .005 | .003 | .292 | 1.722 | .094 |
|  | **current prednisolone dosage** | .000 | .002 | .036 | .212 | .833 |
| a. Dependent Variable: CCA PI | | | | | | |

| **Model** | | **Unstandardized Coefficients** | | **Standardized Coefficients** | **t** | **Sig.** |
| --- | --- | --- | --- | --- | --- | --- |
|  |  | **B** | **Std. Error** | **Beta** |  |  |
| **1** | **(Constant)** | 1.064 | .431 |  | 2.469 | .020 |
|  | **cranial/overlap** | .746 | .331 | .406 | 2.249 | .033 |
|  | **disease duration** | .003 | .004 | .151 | .798 | .432 |
|  | **current prednisolone dosage** | -.001 | .002 | -.049 | -.260 | .797 |
| a. Dependent Variable: CCA PI | | | | | | |

| **Model** | | **Unstandardized Coefficients** | | **Standardized Coefficients** | **t** | **Sig.** |
| --- | --- | --- | --- | --- | --- | --- |
|  |  | **B** | **Std. Error** | **Beta** |  |  |
| **1** | **(Constant)** | 1.173 | .673 |  | 1.742 | .107 |
|  | **extracranial/overlap** | .338 | .437 | .190 | .772 | .455 |
|  | **disease duration** | .020 | .011 | .500 | 1.891 | .083 |
|  | **current prednisolone dosage** | .014 | .011 | .340 | 1.295 | .220 |
| a. Dependent Variable: CCA PI | | | | | | |

*CCA: common carotid artery, PI: pulsatility-index*

**Tables S12.** Regression analyses of CCA RI adjusted for phenotype, disease duration, and glucocorticoid dose.

| **Model** | | **Unstandardized Coefficients** | | **Standardized Coefficients** | **t** | **Sig.** |
| --- | --- | --- | --- | --- | --- | --- |
|  |  | **B** | **Std. Error** | **Beta** |  |  |
| **1** | **(Constant)** | .743 | .068 |  | 10.876 | <.001 |
|  | **cranial/extracranial** | .021 | .044 | .080 | .482 | .633 |
|  | **disease duration** | .001 | .001 | .302 | 1.751 | .089 |
|  | **current prednisolone dosage** | 7.453E-5 | .000 | .026 | .151 | .881 |
| a. Dependent Variable: CCA RI | | | | | | |

| **Model** | | **Unstandardized Coefficients** | | **Standardized Coefficients** | **t** | **Sig.** |
| --- | --- | --- | --- | --- | --- | --- |
|  |  | **B** | **Std. Error** | **Beta** |  |  |
| **1** | **(Constant)** | .652 | .105 |  | 6.234 | <.001 |
|  | **cranial/overlap** | .129 | .080 | .301 | 1.607 | .120 |
|  | **disease duration** | .001 | .001 | .165 | .844 | .407 |
|  | **current prednisolone dosage** | .000 | .001 | -.046 | -.239 | .813 |
| a. Dependent Variable: CCA RI | | | | | | |

| **Model** | | **Unstandardized Coefficients** | | **Standardized Coefficients** | **t** | **Sig.** |
| --- | --- | --- | --- | --- | --- | --- |
|  |  | **B** | **Std. Error** | **Beta** |  |  |
| **1** | **(Constant)** | .601 | .138 |  | 4.356 | <.001 |
|  | **extracranial/overlap** | .088 | .090 | .240 | .984 | .345 |
|  | **disease duration** | .004 | .002 | .496 | 1.892 | .083 |
|  | **current prednisolone dosage** | .002 | .002 | .291 | 1.115 | .287 |
| a. Dependent Variable: CCA RI | | | | | | |

CCA: common carotid artery, RI: resistance-index

**Tables S13.** Results of the Propensity Score Matching analysis.

| **Variable** | **SMD_before** | **abs_SMD_before** | **type** | **SMD_after** | **abs_SMD_after** |
| --- | --- | --- | --- | --- | --- |
| **Age** | 1,156 | 1,156 | continuous | -0,360 | 0,360 |
| **Hypertension** | 0,467 | 0,467 | binary | -0,286 | 0,286 |
| **BMI** | -0,433 | 0,433 | continuous | -0,361 | 0,361 |
| **Diabetes** | 0,349 | 0,349 | binary | 0,000 | 0,000 |
| **Nicotine use** | -0,165 | 0,165 | continuous | -0,218 | 0,218 |
| **Sex** | 0,032 | 0,032 | binary | -0,348 | 0,348 |

BMI: body-mass-index*, SMD_ before: standardized mean difference before* Propensity Score Matching *(PMS), SMD__after: standardized mean difference after PMS, abs_SMD: absolut standardized mean difference*

The results show that before matching, there were substantial imbalances between groups, as indicated by large standardized mean differences (SMDs), along with notable imbalances across the other covariates. After matching the SMDs were markedly reduced for the six CV covariates (age, hypertension, BMI, diabetes, nicotine, sex), indicating improved balance across. Overall, the post-matching SMDs are mostly below commonly used thresholds (≈0.1–0.5), suggesting that the matching procedure was effective in achieving comparable groups.
